# Changes in Soil Carbon and Nitrogen following Land Abandonment of Farmland on the Loess Plateau, China

**DOI:** 10.1371/journal.pone.0071923

**Published:** 2013-08-05

**Authors:** Lei Deng, Zhou-Ping Shangguan, Sandra Sweeney

**Affiliations:** 1 State Key Laboratory of Soil Erosion and Dryland Farming on the Loess Plateau, Northwest A&F University, Yangling, Shaanxi, China; 2 Institute of Environmental Sciences, University of the Bosphorus, Istanbul, Turkey; DOE Pacific Northwest National Laboratory, United States of America

## Abstract

The revegetation of abandoned farmland significantly influences soil organic C (SOC) and total N (TN). However, the dynamics of both soil OC and N storage following the abandonment of farmland are not well understood. To learn more about soil C and N storages dynamics 30 years after the conversion of farmland to grassland, we measured SOC and TN content in paired grassland and farmland sites in the Zhifanggou watershed on the Loess Plateau, China. The grassland sites were established on farmland abandoned for 1, 7, 13, 20, and 30 years. Top soil OC and TN were higher in older grassland, especially in the 0–5 cm soil depths; deeper soil OC and TN was lower in younger grasslands (<20 yr), and higher in older grasslands (30 yr). Soil OC and N storage (0–100 cm) was significantly lower in the younger grasslands (<20 yr), had increased in the older grasslands (30 yr), and at 30 years SOC had increased to pre-abandonment levels. For a thirty year period following abandonment the soil C/N value remained at 10. Our results indicate that soil C and TN were significantly and positively correlated, indicating that studies on the storage of soil OC and TN needs to focus on deeper soil and not be restricted to the uppermost (0–30 cm) soil levels.

## Introduction

Changes in land use have important effects on regional ecological processes and global climate change [1]–[2]. During the past two decades, many studies have focused on the effects of land-use change on soil organic carbon (SOC) and total nitrogen (TN) in terrestrial ecosystems [3]–[5]. The large differences in climatic conditions [6], soil properties [7], and type of land use change [8] are three factors whose effects are not yet well understood.

The revegetation of degraded land is one of the principal strategies for the control of both soil erosion and ecosystem recovery in fragile regions where either anthropogenic activities or severe environmental conditions can lead to disturbance [9]. Revegetation also greatly influences soil quality, C and N cycling, land management, as well as regional socioeconomic development [10]–[11]. The development of managed grassland and forest accelerates ecosystem restoration [12], affecting C and N cycles and C and N pools stored in soils [10], [13]. Altered C and N cycles and C and N pools influence the production of biomass and ecosystem function [14]. Furthermore, C–N interactions are very important in determining whether the C sink in land ecosystems can be sustained over the long term [15]–[16]. Luo et al. [15] have proposed that N dynamics are a key factor in the regulation of long-term terrestrial C sequestration. N may become progressively more limiting as C accumulates in ecosystems under elevated CO_2_ if the total N content in an ecosystem does not change [15]. Therefore, studying the dynamics of organic carbon (OC) and N in soils along a restoration succession gradient and analyzing the relationships between C and N storage dynamics following restoration may be of importance in improving our knowledge of the sustainable management of land resources and predictions of future global C and N cycling.

Large-scale monocropping and over-grazing [17] have affected the semi-arid northern Loess Plateau in China. In addition, over the last century, the expanding human population, combined with a changing lifestyle, has accelerated ecosystem fragmentation and degradation [9]. To stabilize the fragile natural ecosystems characteristic of the Loess Plateau and to alleviate the degradation of land, the Chinese government has launched a series of nationwide conservation projects. One such project converts degraded farmland to either grassland or forest [18]–[19] to control soil erosion, increase storage of SOC and N, and prevent the occurrence of soil desiccation on the Loess Plateau [11]. Restoration succession may affect SOC and N decomposition. With regard to these ecosystem functions, the retention of C and N in soil is crucial [20]. This is of particular concern at sites with substantial N saturation, which are becoming increasingly widespread due to elevated atmospheric N deposition.

Previous studies report that the revegetation of degraded land can increase OC and N storages in soil [9], [13]. Wang et al. [21] reported that both SOC and TN increase as a linear function of years abandoned. However, many previous studies have focused primarily on the topsoil (0–30 cm) [21]–[24]. Little is known about long-term changes to SOC and N in the deeper soil layers of the restoration succession on the Loess Plateau. This information can be useful in estimating the temporal distribution of storage of SOC and N and for evaluating OC and N dynamics throughout the conversion from managed to natural communities in semi-arid regions.

The objectives of this work were to investigate changes in SOC and TN concentration, soil OC and N storage and the relationship between SOC and TN with time since abandonment of farmland with depth in the soil profile.

## Materials and Methods

### Site description

**Figure 1:**
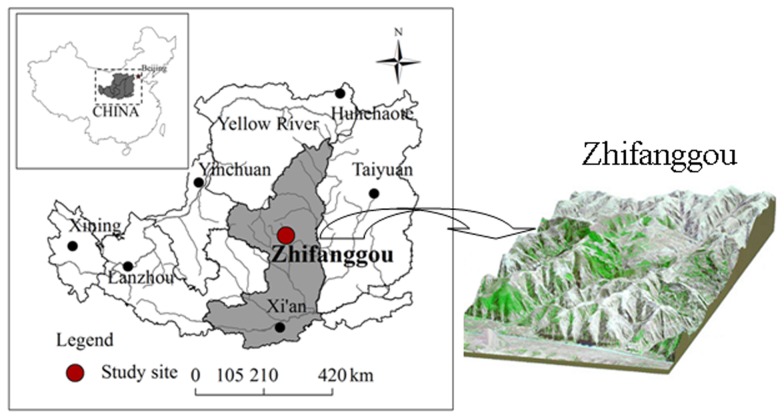
Location and DEM model of the Zhifanggou watershed (adapted from Wang et al., 2011).

**Figure 2:**
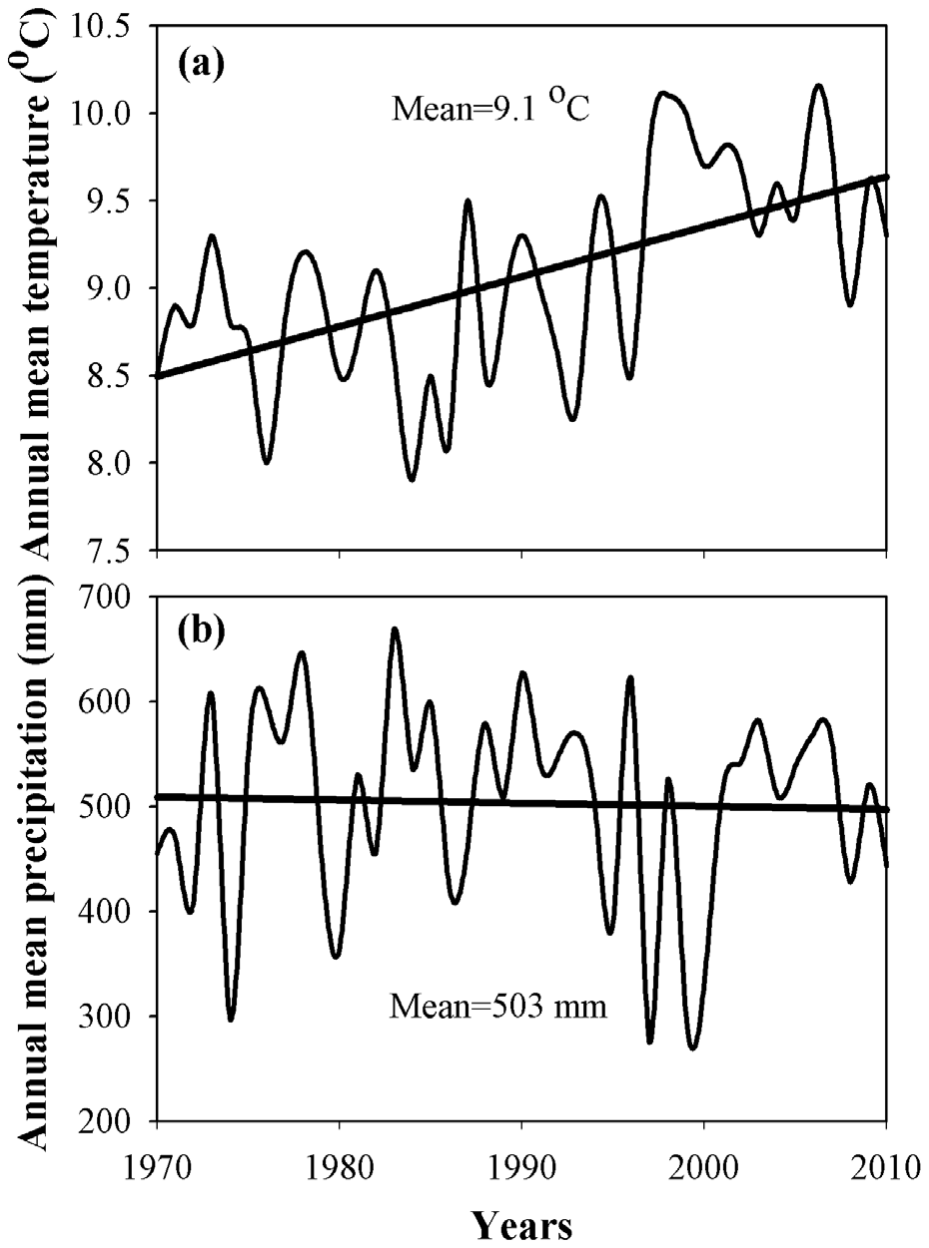
Annual mean temperature (a) and Annual mean precipitation (b) at the study site 1970–2010. Note: the solid lines in the figures a, b are the dynamic fitting curve for annual mean temperature, annual mean precipitation changes with time.

This study was conducted in the Zhifanggou watershed in Ansai County, Shaanxi Province, NW China (36°46′28″–36°46′42″N, 109°13′03″–109°16′46″E; 1,010–1,431 m a.s.l., 8.27 km^2^) (Figure 1). The study area is characterized by a semi-arid climate and a deeply incised hilly-gully Loess landscape. Slopes vary between 0°–65°. The mean annual temperature range is 9.1±0.1°C from 1970 to 2010, and the annual mean temperature has increased over time (Figure 2a), in summer, the highest temperature is 35.3±2.3°C and in winter, the lowest temperature is −20.3±3.7°C from 1970 to 2010; the average frost-free period is 157 days. Mean annual precipitation is 503±15 mm (from 1970 to 2010) (Figure 2b), of which 70% falls between July and September. The loess-derived soils are fertile but extremely susceptible to erosion. The sand, silt and clay contents are 65%, 24% and 11%, respectively [21]. The main grassland species are *Stipa bungeana*, *Bothriochloa ischaemum*, *Artemisia sacrorum*, *Potentilla acaulis*, *Stipa grandis*, *Androsace erecta*, *Heteropappus altaicus*, *Lespedeza bicolor*, *Artemisia capillaries*, and *Artemisia frigid*, of which, *S. bungeana* is the most widely distributed. In addition, shrubland species such as *Rosa xanthina*, *Spiraea pubescens*, and *Hippophae rhamnoides* can be found in gullies. The primary planted trees in the study area are *Robinia pseudoacacia*, *Populus simonii*, *Caragana microphylla*, and *Platycladus orientalis*.

### Experimental design and sampling

A common method used to study vegetation restoration is to monitor plants and soils under similar climatic conditions following the sequence of vegetation development [25]. This chronosequence method is widely adopted in applied ecosystem research [26]–[27] and is considered a “retrospective” research method because it compares existing conditions with original conditions and treatments [28]. The substitution of “space” for “time” is an effective way of studying change over time [28]–[29]. It makes the critical assumption that each site in the sequence differs only in age and that each site has traced the same history in both its abiotic and biotic components [27]. Before revegetation soil conditions are largely driven by geomorphological processes. Thus, sites stabilized through revegetation for different periods of time offer an ideal opportunity to understand vegetation succession processes in extreme environments.

An abandoned farmland chronosequence in the watershed was selected for study after the history of the sites was determined through interviews with local farmers (Mr. Haibin Zhang, Soil and Water Conservation Experiment Station, Northwest A&F University, Ansai County, Shaanxi, NW China). Five age classes, 1, 7, 13, 20, and 30 years were selected. In August 2011, when the grassland community biomass peaked, five sites were established in each of the age classes, and the sites were separated by 0.5–1.5 km apart. At each site we set up a plot of 20 m×20 m. In each plot, five quadrats (1 m×1 m) were separately chosen in each of the four corners and center of the plot. In total, we surveyed five plots with twenty-five quadrats in each age class, and twenty-five plots with one hundred and twenty-five quadrats for our study. In each quadrat, the coverage and height, above- and belowground biomass, litter biomass, and soil samples in 0–100 cm soil cores were observed. The morphological traits of the herbage in each age group are listed in Table 1. The plots were all located near the top of loess mounds and there was little difference among the sites in altitude (1185–1341 m), aspect (half south-facing and half north-facing), gradient (18°–37°), or previous farming practices. In the study area, the soils were loess-derived. In addition, five sites on maize farmland (CK) were selected for comparison. Before the farmland was abandoned maize (*Zea mays*) had been widely seeded. The average amount of fertilizer applied was 225–300 kg ha^−1^ of sheep manure as the base fertilizer in April and 300–450 kg ha^−1^ of urea which was applied in June as topdressing. We state clearly that no specific permissions were required for the location. We confirm that the location is not privately-owned or protected in any way. We confirm that the field studies do not involve endangered or protected species.

**Table 1:** Dominant species and their biomass, total cover, height at different number of years abandoned.

Years aban- doned (yr)	Above- ground biomass (g m^−2^)	Below- ground biomass (g m^−2^)	Accumulative litter biomass (g m^−2^)	Coverage (%)	Height (cm)	Dominant species
1	169.34±49.67a	78.92±13.57c	28.90±4.26c	8.8±0.58c	66±6.96ab	*Artemisia scoparia*
7	73.73±7.96a	138.73±6.72c	118.51±14.93bc	19.4±2.86bc	41.6±7.88b	*Lespedeza bicolor*+ *Setaira viridis*
13	100.26±19.01a	218.71±36.29c	130.78±33.00bc	24.4±5.61abc	68.2±6.01a	*Agropyron cristatum*+ *Heteropappus altaicus*
20	210.42±56.73a	575.21±129.16b	209.79±35.99ab	41±10.77ab	69±5.79a	*Artemisia sacrorum*+ *Bothriochloa ischaemum*
30	159.08±12.03a	1080.34±111.18a	281.86±25.77a	48±6.04a	52±2.07ab	*Bothriochloa ischaemum*+ *Stipa bungeana*

Different letters indicate significant differences at *P*<0.05 among years abandoned. Values are mean ±SE of 5 sites.

In each quadrat, all the aboveground parts of the green plants were cut, collected from the ground and put into envelops and tagged, as was all litter. Because the biomass samples were large, they were weighed fresh and then a part of each sample was dried and weighed. The aboveground biomass of the samples was calculated by multiplying the ratio of the dry weight/fresh weight ratio by the fresh weight.

Soil samples were taken at five points in the quadrats of each plot. These were the four corners and center of the biomass sampling sites as described above. Litter horizons were removed before soil sampling. Soil sampling, using a soil drilling sampler (9 cm inner diameter), was done in seven soil layers, 0–5, 5–10, 10–20, 20–30, 30–50, 50–70, and 70–100 cm. We then mixed the same layers together to make one sample. All samples were sieved through a 2 mm screen, and roots and other debris were removed. Each sample was air-dried and stored at room temperature for the determination of soil physical and chemical properties. The soil bulk density (g cm^−3^) of the different soil layers was measured using a soil bulk sampler with a 5 cm diameter and 5 cm high stainless steel cutting ring (5 replicates) at points adjacent to the soil sampling quadrats. The original volume of each soil core and its dry mass after oven-drying at 105°C were measured.

To measure belowground biomass, soil sampling was done three times in seven soil layers, 0–5, 5–10, 10–20, 20–30, 30–50, 50–70, and 70–100 cm at a depth of 0–100 cm in each quadrat using a 9 cm diameter root auger. The majority of the roots were found in the soil samples thus obtained and then isolated using a 2 mm sieve. The remaining fine roots taken from the soil samples were isolated by spreading the samples in shallow trays, overfilling the trays with water and allowing the outflow from the trays to pass through a 0.5 mm mesh sieve. No attempts were made to distinguish between living and dead roots. All the roots thus isolated were oven-dried at 65°C and weighed to within 0.01 g.

### Physical and chemical analysis

Soil bulk density (BD) was calculated depending on the inner diameter of the core sampler, sampling depth and the oven dried weight of the composite soil samples [30]. Soil OC content was assayed by dichromate oxidation [31] and soil TN content was assayed using the Kjeldahl method [32].

### Calculation of soil C and N storages

Our sample soils did not have any coarse fraction (>2 mm). Therefore, the study used the following equation to calculate soil organic carbon storage (Cs) [22]:
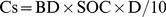
(1)in which, Cs is SOC storages (Mg ha^−1^); BD is soil bulk density (g cm^−3^); SOC is soil organic carbon concentration (g kg^−1^); and D is soil thickness (cm).

The following equation was used to calculate soil N storage (Ns) [33]:
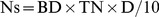
(2)in which, Ns is soil N storage (Mg ha^−1^); BD is soil bulk density (g cm^−3^); TN is soil TN concentration (g kg^−1^); and D is soil thickness (cm).

### Statistical analysis

One-way ANOVA was used to analyze the means of the same soil layers among the different abandoned years. Differences were evaluated at the 0.05 significance level. When significance was observed at the *P*<0.05 level, Tukey's post hoc test was used to carry out the multiple comparisons. All statistical analyses were performed using the software program SPSS, ver. 17.0 (SPSS Inc., Chicago, IL, USA).

## Results

### Dynamics of SOC and TN

**Figure 3:**
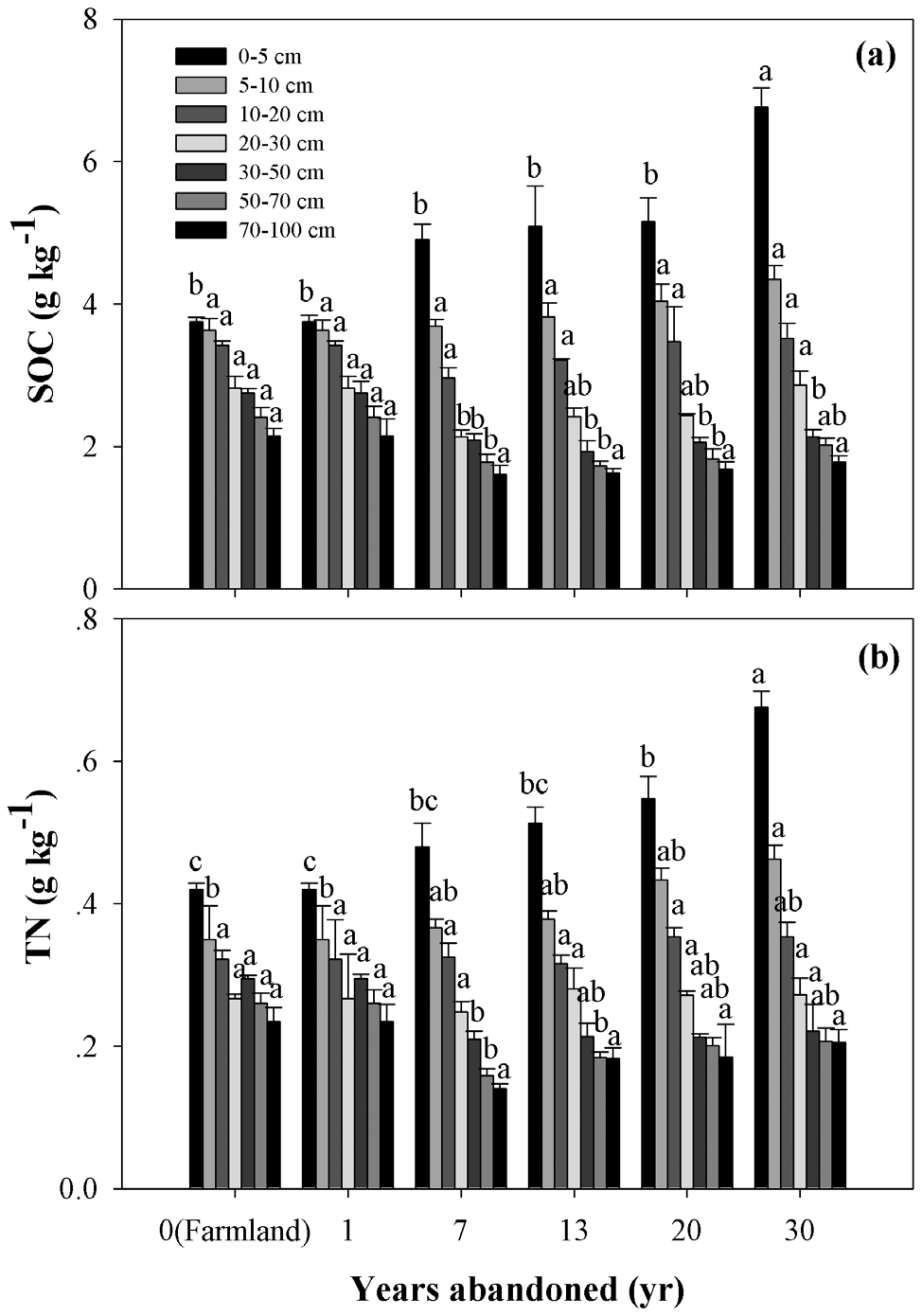
SOC and TN dynamics for seven soil layers of 0–100 cm following the conversion of farmland to grassland. Values are in the form of Mean ± SE and the sample size n = 5. Different lower-case letters above the bars mean significant differences in the same soil layers among abandoned land in different years (*P*<0.05).

SOC in 0–5 cm soil was higher in older grassland following abandonment (Table 2, Figure 3a). In the first 20 years, 0–5 cm SOC showed no significant changes; after 20 years, it had significantly increased (Figure 3a). The 5–20 cm SOC had not significantly increased (Table 2). The 20–70 cm SOC was significantly lower in the younger grasslands (<20 yr) and higher in the older grasslands (30 yr) (Figure 3a). When abandoned for 30 years, 20–70 cm SOC had increased to the level prior to abandonment (maize) (Figure 3a). The 70–100 cm SOC showed no significant changes since having been abandoned, but also showed a tendency to be low in younger grasslands and higher in the older grasslands (Table 2, Figure 3a).

**Table 2:** One-way analysis of variance (ANOVA) for soil properties in the seven soil layers among abandoned farmland in different years.

Soil layer (cm)	Df	SOC		TN	
		F	sig. (*P*)	F	sig. (*P*)
0–5	5	10.367	0.0001**	14.557	0.0001**
5–10	5	2.604	0.0668	3.651	0.0217*
10–20	5	0.88	0.4936	0.397	0.8081
20–30	5	5.322	0.0044**	0.132	0.9688
30–50	5	6.892	0.0012**	3.348	0.0298*
50–70	5	5.787	0.0029**	6.963	0.0011**
70–100	5	2.567	0.0697	1.825	0.1636

Soil depth (cm)	Df	C storages		N storages	
		F	sig. (*P*)	F	sig. (*P*)
0–5	5	5.71	0.0031**	5.809	0.0029**
0–10	5	4.053	0.0145*	4.527	0.0091**
0–20	5	0.783	0.5496	0.732	0.5811
0–30	5	1.404	0.2688	0.139	0.966
0–50	5	4.483	0.0095**	1.014	0.4237
0–70	5	7.042	0.0010**	3.089	0.0392*
0–100	5	7.905	0.0005**	3.783	0.0190*

* indicates significant at *P*<0.05 and

** **indicates significant at *P*<0.01.

Soil TN in 0–5 cm soil was also higher in older grassland following abandonment (Table 2, Figure 3b). In the first 13 years after having been abandoned, TN in the 0–5 cm soil layer showed no significant changes; after 13 years, it had significantly increased (Figure 3b). The TN in the 5–10 cm soil layer showed no significant changes in the first 13 years, whereas after 13 years, it had significantly increased (Figure 3b). The 10–30 cm soil TN did not increase significantly (Table 2, Figure 3b). The 30–70 cm soil TN first decreased significantly and then increased to the level prior to abandonment (maize) (Figure 3b). Similar to the SOC, 70–100 cm soil TN had showed no significant changes since abandonment, but also showed a tendency to be lower in younger grasslands and higher in older grasslands (Table 2, Figure 3b).

### C and N storages dynamics

**Figure 4:**
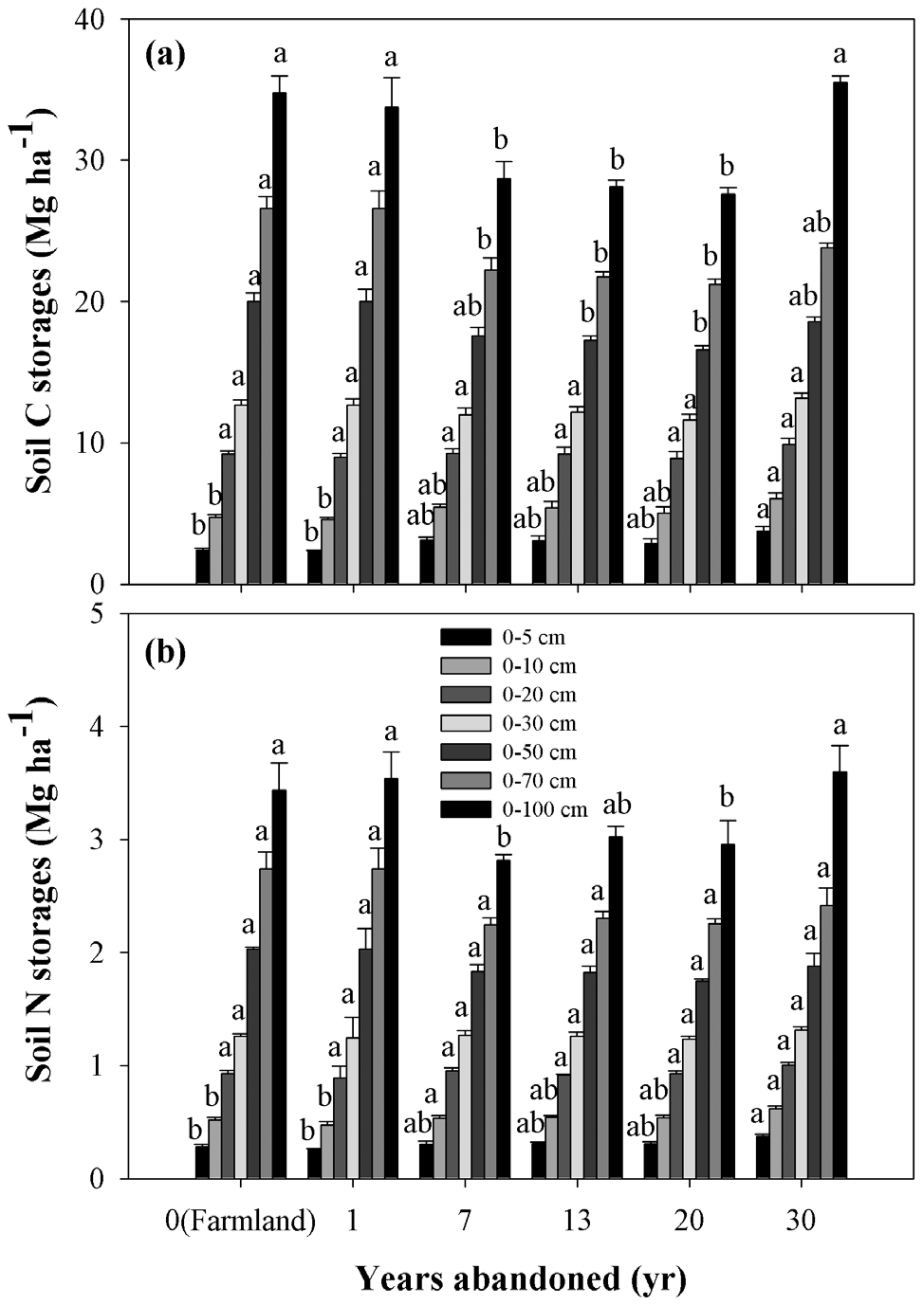
Dynamics of soil OC and N storages in 0–100 cm soil depth following the conversion of farmland to grassland. Values are in the form of Mean ± SE and the sample size n = 5. Different lower-case letters above the bars mean significant differences in the same soil layers among abandoned land in different years (*P*<0.05).

Soil OC storage in the 0–10 cm soil was also higher in older grassland since abandonment (Table 2, Figure 4a). After having been abandoned for 30 years, the 0–10 cm soil OC storage had increased significantly. 0–30 cm soil OC storage had not increased significantly (Table 2, Figure 4a). 0–100 cm soil OC storage was significantly lower in the younger grasslands (<20 yr) and higher in the older grasslands (30 yr); at 30 years it had increased to pre-abandonment levels (Figure 4a). Soil C storage changed mainly in the top 10 cm of soil following the conversion of farmland to grassland for a period of 30 years.

Similar to the accumulative soil OC storage, soil N storage had changed mainly in the top 10 cm soil layer 30 years after having been abandoned. The conversion of farmland into grassland significantly increased the soil N storage in the 0–10 cm soil (Table 2, Figure 4b). After having been abandoned for 30 years, 0–10 cm soil N storage had significantly increased. 0–50 cm soil N storage had not significantly increased (Table 2, Figure 4b). 0–100 cm soil N storage was also significantly lower in younger grasslands (<20 yr) and higher in older grasslands (30 yr), until after thirty years it had increased to pre-abandonment levels (maize) (Figure 4b).

### Relationship between Soil C and N

**Figure 5:**
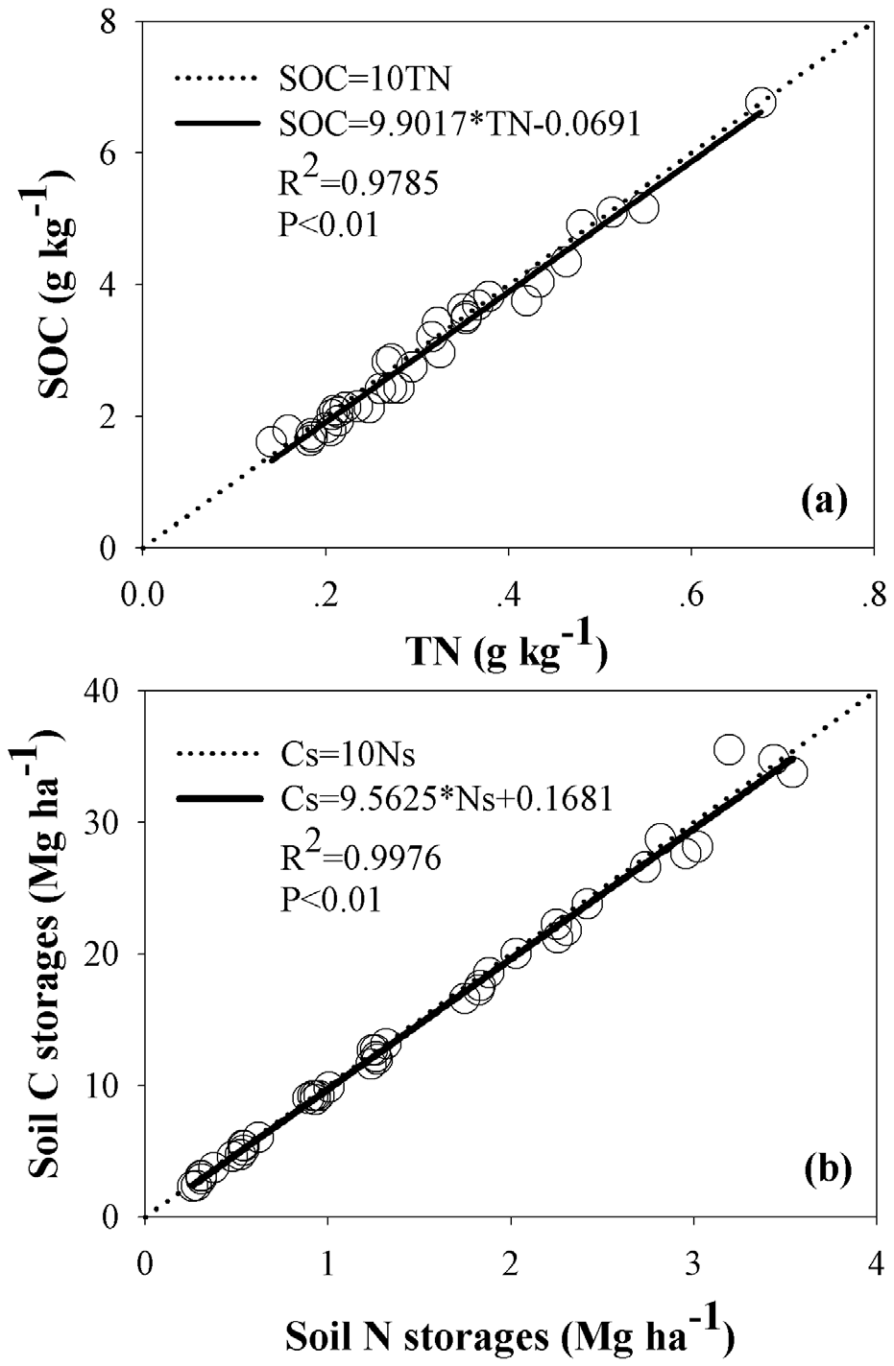
Relationship between SOC and TN, soil OC storages and soil N storages, and accumulative soil C storages and soil N storage.

Soil C and N showed significant positive correlations (Figure 5). The relationship between SOC and TN, soil C storage and soil N storage were significant (*P*<0.01). In the process of the revegetation, soil OC and TN, soil C storage and N storage approximately represents SOC = 10TN (Figure 5a) and Cs = 10Ns (Figure 5b).

## Discussion

**Figure 6:**
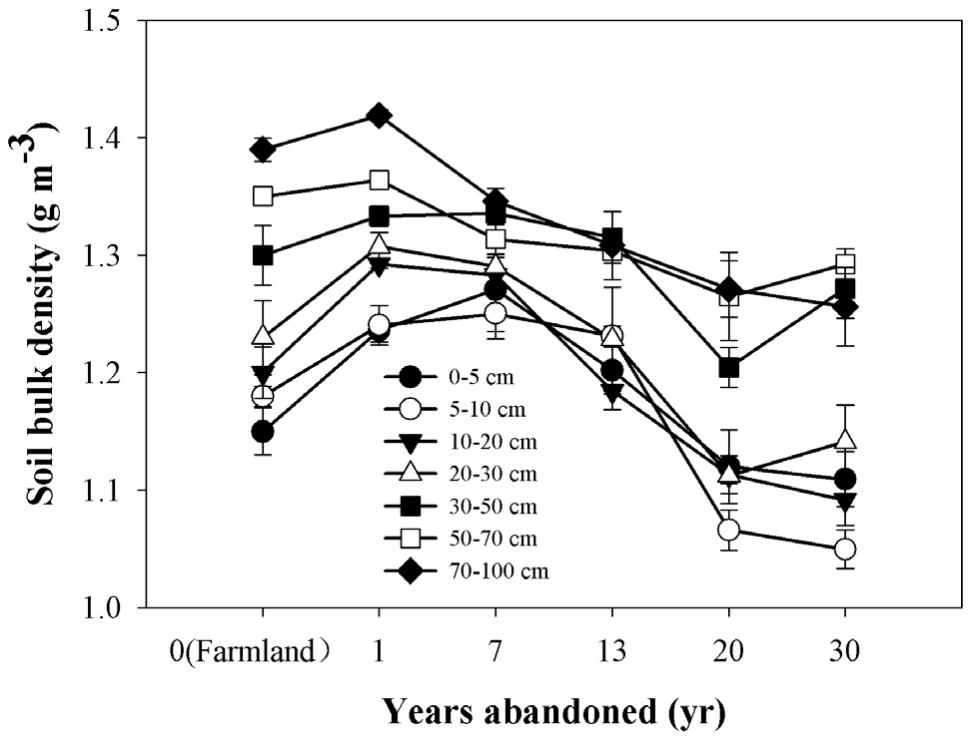
Dynamics of soil bulk density in 0–100 cm soil depth following the conversion of farmland to grassland. Values are in the form of Mean ± SE and the sample size n = 5.

The soil C and N results supported the hypothesis that soil organic C and N conditions in both the top soil and in the deeper soil layer are significantly affected by land use change on the northern Loess Plateau. In our study, top soil OC and TN were higher in older post-abandonment grassland, especially in the 0–5 cm soil depth (figure 3), indicating the accumulation of soil OC and TN by revegetation [23]–[24]. These results agree with those of Wang et al. [21], who studied changes to the physico-chemical properties of top soil during natural succession on abandoned farmland in the Zhifanggou watershed. The evident increase may be partly attributed to a lower fraction of non-soluble materials in more readily decomposed plant residues. In the farmland, cultivation breaks up soil aggregates, decreases total soil porosity, and accelerates composition and mineralization of soil organic matter (SOM) due to exposure of previously accessible SOM to microbial attack [34]. This results in a reduction in the amounts of intra-aggregate light fraction organic carbon (LFOC) and some organomineral SOC [35]. In addition, the reduction of crop residue return to soil may also be a factor as farmers take away straw with grain harvesting each year. This speculation can be supported by the results of Wu et al. [36] in this region where plant residue in the top soil layer was reduced considerably after native grasslands were cultivated, contributing to the decrease in LFOC and SOC. Conversely, the conversion of farmland into grassland increases SOC and its fractions [37], and increases total soil porosity, thus resulting in a reduction in soil BD (Figure 6, [21]).

**Figure 7:**
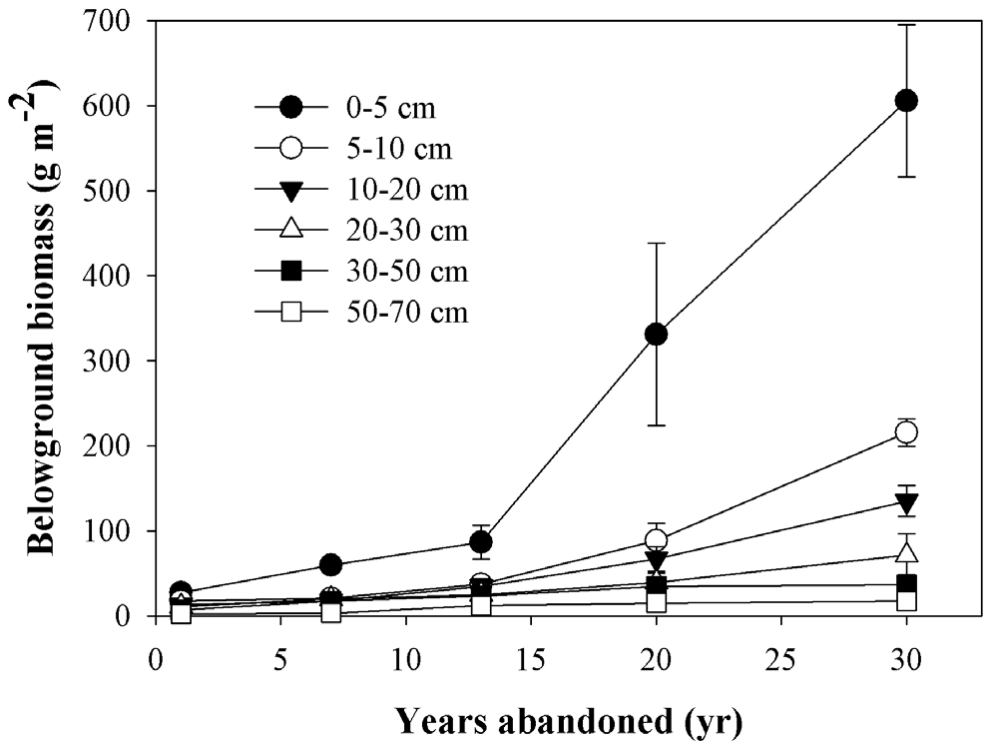
Dynamics of belowground biomass in 0–100 cm soil depth following the conversion of farmland to grassland. Values are in the form of Mean ± SE and the sample size n = 5. It didn't have roots in the 70–100 cm soil layers.

Aboveground vegetation plays an important role in regulating the biogeochemistry of ecosystems by fixing C and nutrients and preventing the loss of nutrients under disturbance, such as plant decline, acid-rain and climate change [38]. It is also clear from these results that revegetated grassland has a great impact on the storage of soil OC and N. In our study, both soil OC and N storage in the 0–10 cm soil depth was higher in older grassland that had been abandoned for thirty years (Figure 4). This indicates that during the 30 year period soil OC and N storage mainly had changed in the top 10 cm of soil since abandoned. There may be a range of potential mechanisms through which soil OC and N in the top soil increased with revegetation. A prime candidate is the return of both C and N from increased aboveground biomass and litter. As soil OC and N input are mainly derived from the decomposition of litter [21], primary productivity is the main driver of soil carbon sequestration [39], which resulted primarily in soil OC and N storage increasing in the top soil. Secondly, belowground biomass (dead roots, mycorrhizae, and exudates) is an important element of soil carbon sequestration [40]. Belowground biomass increased in the time after the farmland had been abandoned (Table 1, Figure 7). Thirdly, changes in vegetation composition and the dominant plant functional group could affect the sequestration of C and N in the soil [39], [41]. Plant functional composition strongly influences the chemical and physical composition of litter inputs, and thereby their decomposability, carbon loss through soil respiration and leaching, and carbon immobilization in hummified plant residues [39], and the increase of C3 plants can increase soil C and N accumulation in meadow soils [41]. Revegetation had a direct effect on the dominant vegetation species, vegetation cover, height, above- and belowground biomass (Table 1).

SOC and TN at deeper soil levels (>30 cm) were lower in younger grasslands and higher in older grasslands (Figure 3), this is probably due to long-term natural organic fertilizer and inorganic fertilizer inputted into the soil resulting in higher SOC and N in the farmland stage. So, this initial loss of soil OC and N following the conversion of farmland has been commonly attributed to the net effect of decreased organic matter inputs and losses through decomposition [42]. During the course of grasslands development, we observed an increase in the soil SOC and N storage, after 30 years of revegetation, where deeper soil C and N had returned to pre-abandonment levels (Figure 3); the return of belowground biomass to the deeper soil layer is another reason (Figure 7). Previous studies of soil C dynamics have emphasized the role of physical protection from different particle fractions (sand, silt and clay) [43], microbes and enzymes within aggregates [44], microaggregates and macroaggregates [45], and bacterial and fungal [46], therefore, the mechanism in the soil C and N dynamics in the deeper soil during the process of conversion from farmland into grassland probably relate to those factors. However, our lack of a full understanding of this process calls for more attention to be paid to soil C and N dynamics in the deeper soil layers. Wang et al. [21] found that SOC and TN had a negative relationship with soil bulk density, and Singh et al. [23]–[24] reported that SOC and TN were significantly greater while BD was significantly lower during the restoration of degraded lands, our results agreed with them (Figures 3 and 6). So, we can infer the trend of SOC and TN according to the trend of soil bulk density.

We observed that soil C and N were significantly and positively correlated (Figure 5). In the restoration process, soil OC and TN, soil C storage and N storage approximately represents SOC = 10TN (Figure 5a) and Cs = 10Ns (Figure 5b). So, we can conclude that soil C/N value was 10 in the process of 30 years of conversion from farmland to grassland in the Loess Plateau, a value greater that reported by Liu et al. [35] (C/N = 7.62). Ammonium-N and the sum of NO_3_^−^ -N and NO_2_^−^ -N are the readily available forms of soil N for root uptake. Liu et al. [35] reported that ammonium-N and the sum of NO_3_^−^ -N and NO_2_^−^ -N were lower in grassland than in cropland with the addition of chemical fertilizer N, because the conversion of farmland into grassland increased the C/N ratio and reduced soil mineral N by enhancing soil N immobilization.

Grasslands play an important role in the global C and N cycles [3]–[4], [10]. Grassland in good condition should be in balance in terms of C and N input and output or in the state where C and N input is greater than their output [41]. At least one study has shown that carbon input is greater than carbon output in enclosed grassland [47]. In our study, 0–100 cm soil OC and N storage was significantly lower in the younger grasslands (<20 yr) and higher in the older grasslands (30 yr), at 30 years they had increased to pre-abandonment levels, and that the values in farmland were higher compared to grasslands abandoned for 20 years. Our results indicate that the study of the storage of soil OC and N need to include the deeper soil layer and not focus solely on the top soil. Li et al. [48] also found that SOC density in the deeper soil layer was significantly higher than that of the farmland in the Zhifanggou watershed, Loess Plateau. Li et al. [49] found that soil C and N storage in the deeper layers (mineral layer) show significant difference in top soil layers (organic layer) with time after afforestation at the global scale. Therefore, estimating soil OC and N input or output requires that research consider not only soil depth but also time since abandonment.

## Conclusions

The results of this study indicate that plant succession after land has been abandoned resulted in a significant improvement in the physico-chemical properties of soil. Thirty years following abandonment, soil OC and N storage had increased primarily in the top 10 cm of the soil depth. After 30 years of restoration, deeper soil C and N storage had increased to pre-abandonment levels. This finding indicates that deeper soil has a higher potential to fix both C and N in the future (>30 yr). Thus, in the semi-arid environment of the Loess Plateau, vegetation recovery following abandonment is slow and the improvement of soil properties is likely to require a considerably long period of time (>30 yr). Therefore, the findings are important for assessing the resilience of these degraded ecosystems and developing a more effective strategy of vegetation restoration for the management of degraded grassland from a long-term perspective. More research, for example, on soil physico-chemical properties, soil enzyme activities, soil microbial, animal and plant function and composition, is required to better understand the mechanism behind how the soil fixes C and N in the deeper soil profile of sub-arid regions, for example, the Loess Plateau, China.
