# Correction: Changes in Soil Carbon and Nitrogen following Land Abandonment of Farmland on the Loess Plateau, China

**DOI:** 10.1371/annotation/61b7e0d5-6062-49b7-a270-2c115dd3cb8f

**Published:** 2013-09-04

**Authors:** Lei Deng, Zhou-Ping Shangguan, Sandra Sweeney

There was an error in the Funding statement. The correct version of the statement is available below.

The study was funded by the Strategic Priority Research Program of the Chinese Academy of Sciences (XDA05050403) and the Scholarship Award for Excellent Doctoral Student granted by Ministry of Education. The funders had no role in study design, data collection and analysis, decision to publish, or preparation of the manuscript. 

